# Colorimetric Sensor Arrays Technology in Food Quality and Safety Analysis

**DOI:** 10.3390/mi17070821

**Published:** 2026-07-09

**Authors:** Jie Cao, Ye Qiu, Yang Gao, Guanggui Cheng

**Affiliations:** 1 School of Intelligent Science and Engineering, Jiangsu University, Zhenjiang 212013, China; caojie@ujs.edu.cn; 2 School of Mechanical Engineering, Jiangsu University, Zhenjiang 212013, China; 2222503158@stmail.ujs.edu.cn (Y.Q.); 2222503155@stmail.ujs.edu.cn (Y.G.); 3 Key Laboratory of Intelligent Flexible Actuation and Control in Universities of Jiangsu Province, Jiangsu University, Zhenjiang 212013, China

**Keywords:** colorimetric sensor arrays, food quality assessment, food spoilage detection, adulteration identification, intelligent detection platform

## Abstract

Colorimetric sensor array (CSA) technology has been increasingly applied in food quality and safety analysis due to its advantages of low cost, visual readout, high sensitivity and suitability for on-site monitoring. However, a dedicated review that systematically integrates advances in sensing materials, application scenarios, analytical strategies and practical implementation of CSA technology for real food matrices is still lacking. This review summarizes the fundamental characteristics of CSA technology from the relevant studies published during the past 5 years. The literature was retrieved using keywords such as “colorimetric sensor array” and “food quality detection,” and was filtered by predefined criteria prioritizing original research with verified sensing performance and practical validation in food applications. Emphasis is placed on the design and performance of sensitive materials. Furthermore, using a task-oriented framework, representative studies on CSA applications in food spoilage detection, adulteration identification, food composition analysis, contamination monitoring, food storage monitoring and volatile compound detection are discussed. In addition, the integration of CSA systems with portable devices, machine learning algorithms and intelligent detection platforms is critically analyzed. Finally, the detection characteristics, current challenges and future development prospects of CSA technology in food quality and safety analysis are highlighted. This review is expected to provide valuable insights for further development, optimization and practical implementation of intelligent CSA-based sensing systems in the food industry.

## 1. Introduction

Food quality and safety are closely associated with human health, social stability and the sustainable development of the food industry [1,2]. With the increasing complexity of the global food supply chain, food products are highly susceptible to microbial contamination, oxidative deterioration, chemical residues, and illegal adulteration during production, processing, transportation and storage, which may result in quality degradation and even serious food safety incidents [3]. Therefore, the development of rapid, accurate and highly sensitive detection technologies for food quality assessment is of great significance for ensuring food safety and improving product quality [4]. At present, food quality analysis mainly relies on conventional analytical techniques. Among them, gas chromatography–mass spectrometry (GC-MS), high-performance liquid chromatography (HPLC) and enzyme-linked immunosorbent assays (ELISA) have been widely employed for food composition analysis, contaminant detection and quality evaluation owing to their high sensitivity and reliability [5]. However, these methods generally require expensive instrumentation, complicated sample pretreatment procedures, long analysis times and skilled operators [6], which considerably limit their applicability for rapid on-site monitoring and real-time food inspection. Particularly in food storage, transportation and market circulation processes, traditional analytical methods still face significant challenges in terms of portability, operational simplicity and continuous monitoring capability [7].

With the rapid advancement of sensing technologies, materials science and AI in recent years, colorimetric sensor array (CSA) technology has attracted extensive attention in the field of food quality detection due to its advantages of visual response, low cost, high throughput and operational simplicity [8,9]. A typical CSA system consists of multiple chemically responsive sensing materials with cross-reactive characteristics. Upon interaction with target analytes, distinct color variations are generated and subsequently analyzed through digital imaging and pattern recognition or chemometric algorithms to achieve qualitative and quantitative analysis of complex samples [10,11,12]. Compared with conventional single-sensor systems, CSAs can mimic biological olfactory systems and generate characteristic “fingerprint” responses toward volatile organic compounds (VOCs), biogenic amines, gaseous metabolites and chemical contaminants, thereby enabling highly sensitive and high-throughput food detection [13,14,15,16]. This technology has been validated in a variety of food systems, including vegetables, fruits, cereals, tea and meat [17,18,19,20,21], with applications covering freshness monitoring, spoilage assessment, adulteration identification and contaminant detection [22,23,24,25]. Furthermore, the integration of emerging technologies such as smartphone imaging, machine learning, deep learning and Internet of Things (IoT) platforms has accelerated the development of CSAs toward intelligent, portable and real-time sensing systems, providing new opportunities for on-site rapid detection and digitalized monitoring [26,27,28,29]. These research efforts span from the optimization of sensing materials and image analysis algorithms to the integration of intelligent detection platforms, demonstrating the considerable potential of CSA technology for practical food quality monitoring applications.

Although significant progress has been achieved in the development of colorimetric sensing technologies for food quality and safety detection, previous reviews have broadly addressed electronic noses, electronic tongues, optoelectronic noses and optical colorimetric sensor arrays [30,31,32,33,34,35]. More recent reviews have further examined colorimetric sensing and CSA technology in food quality, safety and authenticity assessment, with emphasis on sensing materials, system designs and representative applications [8,9,36]. Nevertheless, cross-task discussions of the target characteristics, technical requirements and practical constraints associated with different food-analysis applications remain limited. This review adopts a task-oriented framework and organizes representative CSA applications into food spoilage assessment, adulteration identification, food composition and contaminant detection, storage monitoring and volatile-compound analysis. By considering differences in detection targets, sample formats, response signals and data-processing requirements, this review provides a structured reference for identifying potentially relevant sensing strategies without constituting a universal selection rule.

**Figure 1:**
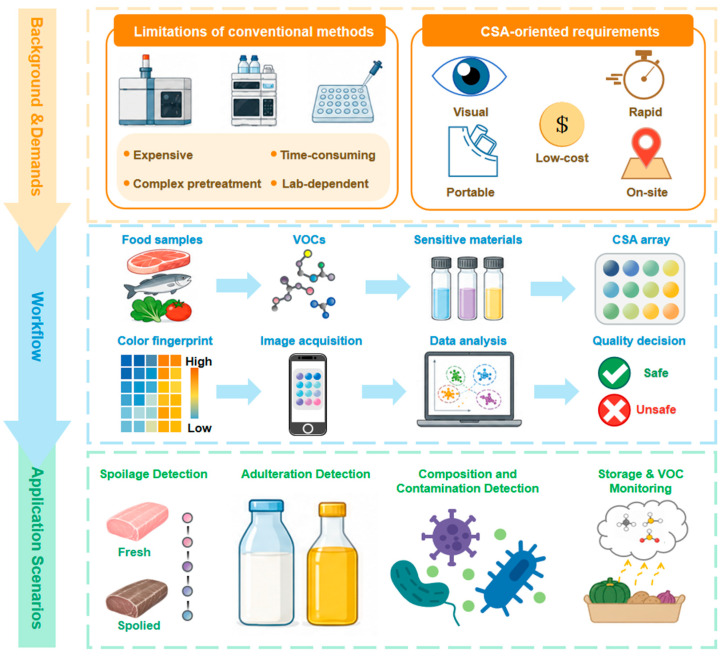
Overall framework of CSA technology for food quality and safety analysis.

As shown in Figure 1, this review presents an overall framework of CSA technology for food quality and safety analysis, organized according to a task-oriented structure covering different application scenarios. First, the fundamental principles of CSA technology, including its working mechanisms and commonly used sensitive materials, are introduced, together with the characteristics and limitations of representative signal-acquisition approaches and material categories. Subsequently, the representative applications and research progress of CSA technology in food quality and safety analysis are discussed from the perspectives of food spoilage detection, food adulteration detection, food composition and contamination detection, as well as food storage and volatile compound detection. Where appropriate, the advantages, limitations and applicability of different sensing strategies are discussed with reference to representative studies and food matrices. Furthermore, the integration of CSA technology with intelligent detection platforms, including smartphone-based image acquisition and pattern-recognition methods [37], is also discussed. Finally, the detection characteristics, existing challenges and future development prospects of CSA technology are summarized, with particular attention to sensor standardization, long-term stability, model transferability, large-scale deployment and the translation of laboratory-scale systems into practical food-monitoring applications.

## 2. Fundamentals of CSA Technology

The origin of colorimetric sensor array (CSA) technology can be traced to the optoelectronic nose system reported by Rakow and Suslick, in which cross-responsive metalloporphyrin dyes enabled odor visualization through characteristic color-change patterns [38]. This strategy was subsequently extended to molecular recognition using chemoresponsive dye arrays, establishing a pattern-based sensing principle distinct from conventional single-receptor detection [39].

### 2.1. Working Mechanism of CSA Technology

**Figure 2:**
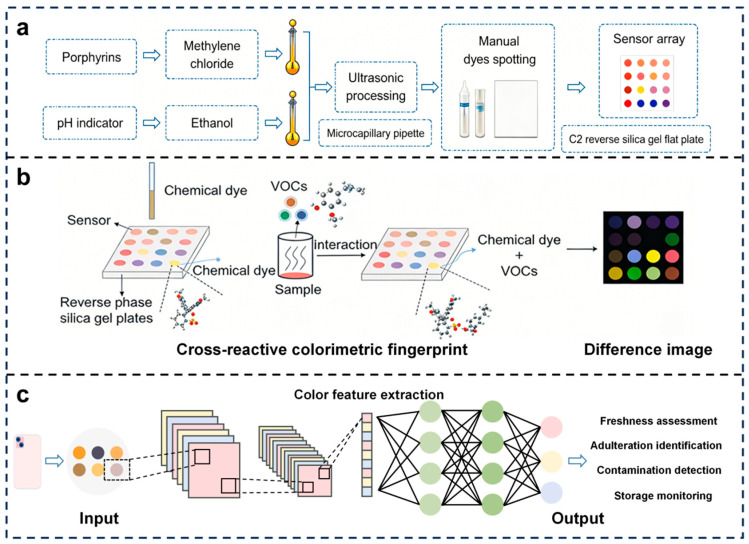
Detection principle and workflow of CSA-based food analysis. (**a**) Fabrication of a chemically diverse sensor array using porphyrins and pH indicators immobilized on a reversed-phase silica gel plate; (**b**) cross-reactive colorimetric responses to food-emitted VOCs and generation of difference images. Reproduced from Zhang et al., 2024 [41]. (**c**) Image acquisition, color feature extraction, and chemometric or learning-based analysis. Reproduced from Zhang et al., 2025 [49].

Colorimetric sensor arrays (CSAs) are cross-responsive optical sensing platforms that convert chemical information from food matrices into colorimetric fingerprints. Unlike conventional single-receptor sensors, CSAs use multiple sensing units with partially overlapping selectivity and identify sample states through the overall color-change pattern of the array. Since food deterioration, fermentation, adulteration, and contamination are usually associated with combined changes in multiple volatile or non-volatile compounds, this sensing mode is highly suitable for food analysis. In this context, chemoresponsive dyes, solid supports, and data processing have been identified as key elements for CSA-based food odor visualization [40]. As shown in Figure 2a, CSA-based food analysis starts with the fabrication of a chemically diverse sensor array, in which porphyrins, metalloporphyrins, pH indicators or other chromogenic probes are immobilized on solid substrates to form spatially separated sensing spots. As illustrated in Figure 2b, VOCs or other food-derived compounds diffuse to the sensing surface and interact with the immobilized dyes through acid–base reactions, metal–ligand coordination, hydrogen bonding, polarity-dependent effects, π-π interactions or other physicochemical processes. Different response preferences of sensing units generate multi-spot color changes, and the difference image obtained before and after exposure can serve as the visual chemical fingerprint of the food sample [41]. However, the cross-reactive nature of CSAs may also cause response overlap and background interference in complex food matrices, while material stability, batch reproducibility, humidity sensitivity, dye leaching and image-acquisition conditions can further affect analytical robustness. To alleviate these limitations, aptamer-assisted nanozyme immunoassays combined with magnetic nanoparticles have been used to improve target recognition and separation efficiency in AFB1 detection [42]. Nanozymes have also been summarized as promising signal-amplification materials for food safety analysis because of their enzyme-like activity, good stability and low cost [43]. In addition, nanobody-based colorimetric-SERS immunosensors can provide dual optical signals to improve the reliability of microcystin-LR detection [44]. Nanozyme-linked colorimetric systems based on the HAuCl_4_/H_2_O_2_ reaction further demonstrate the potential of enzyme-free visual signal amplification [45]. Moreover, Pt-Au nanoflower-based colorimetric-electrochemical platforms offer complementary readouts for antioxidant detection [46]. These strategies suggest that CSA-related colorimetric sensing systems should balance sensitivity, selectivity, stability and practical applicability according to specific food analysis scenarios.

The array images are then collected and analyzed using digital imaging methods. As illustrated in Figure 2c, CSA images are usually acquired by scanners, cameras or smartphones, followed by color feature extraction and chemometric or learning-based analysis. RGB values, grayscale intensity and CIE L*a*b* values are commonly used descriptors because RGB imaging is low-cost, simple and compatible with portable devices. However, RGB-based analysis is susceptible to illumination conditions, camera parameters and background interference. Beyond conventional RGB imaging, multispectral imaging has been integrated with CSA to collect grayscale responses at eight spectral bands for Zhenjiang aromatic vinegar grading and volatile component prediction, showing that spectral acquisition can enrich CSA readouts [47]. Hyperspectral imaging has also been combined with a salt-sensitive AgNPRs-based CSA to acquire spectral information from both salted duck egg samples and the sensor array, enabling quantitative prediction of moisture, lipid, and salt contents during processing [48]. Compared with RGB imaging, multispectral imaging provides richer information at selected wavelengths, whereas hyperspectral imaging offers continuous spectral information for more detailed quantitative prediction. Nevertheless, these advantages are accompanied by higher cost, larger data volume and more complex calibration and modeling procedures. Therefore, RGB imaging is more suitable for low-cost on-site screening, while multispectral and hyperspectral methods are more appropriate for applications requiring higher accuracy or spectral interpretation. The extracted features can then be analyzed by PCA, LDA, SVM or other chemometric and machine learning methods. Deep-learning models have also been applied to CSA image analysis to reduce reliance on manually defined color parameters [49]. Han et al. developed a metal oxide gas sensor array for real-time monitoring of VOC changes during oolong tea oxidation, and the BP-ANN model achieved a prediction accuracy of 94.11%, supporting the value of array-based signal acquisition and data-driven analysis in food process monitoring [50]. Overall, CSA can be regarded as an integrated workflow covering sensing mechanisms, array design, signal acquisition, data interpretation and food applications, in which the selection of imaging and readout strategies should balance sensitivity, selectivity, portability, cost and data-processing complexity according to specific food analysis scenarios [9].

### 2.2. Sensitive Materials of CSA Technology

The above workflow indicates that CSA-based food analysis is essentially a material-response-driven pattern recognition process. The quality of the colorimetric fingerprint depends not only on image acquisition and data interpretation, but also on the chemical diversity, response stability and interfacial compatibility of the sensing units. Since different sensitive materials determine the types of analyte–material interactions, response intensity and array reproducibility, material design serves as the core basis for constructing reliable CSA systems. Therefore, the following section summarizes the representative sensitive materials used in food-related CSAs and their corresponding colorimetric response mechanisms. The color response of a CSA is closely related to the sensing material selected for each array spot. The materials used in food-related CSAs can be grouped into organic-based and inorganic-based systems. Organic materials include chemoresponsive dyes, organic nanoparticles, functional polymers and immunoassay-related platforms, while inorganic materials include AuNPs, AgNPs, nanozymes, MNPs and MOFs [51]. Their colorimetric responses arise from different mechanisms, including molecular structural variation, aggregation, plasmonic shifts, catalytic reactions, etching, magnetic enrichment, and porous adsorption. To provide a clearer comparison of different sensing material classes, Table 1 summarizes their representative response mechanisms/functions, advantages, limitations and related references.

**Figure 3:**
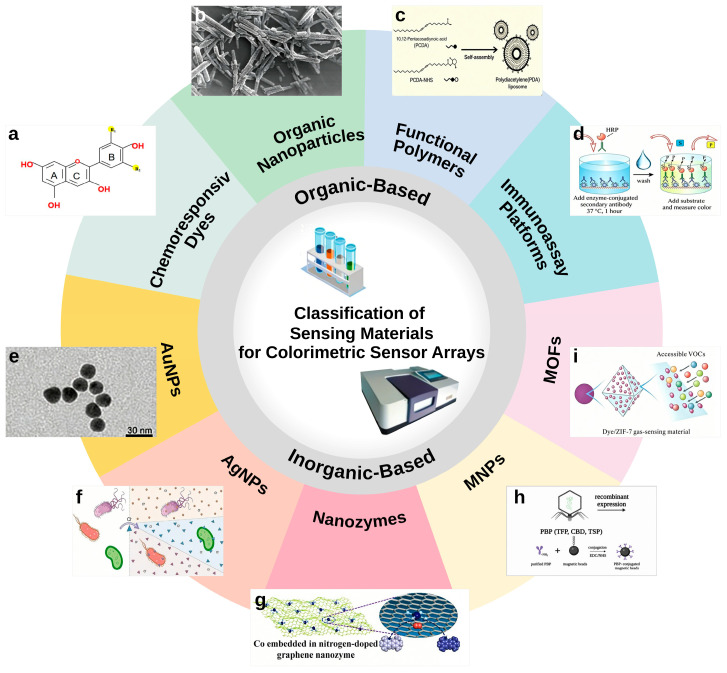
Representative organic- and inorganic-based sensing materials of CSA technology. (**a**) Natural anthocyanin pigment array for AFB1-related wheat quality assessment. Reproduced from Jiang et al., 2025 [52]. (**b**) Flavonol nanocrystal-based packaging film for banana preservation and freshness indication. Reproduced from Li et al., 2023 [53]. (**c**) Polydiacetylene hydrogel beads for biogenic amine detection in spoiled meat. Reproduced from Jang et al., 2023 [54]. (**d**) Lateral flow immunoassay with magnetic enrichment and AuNP-based signal amplification for paraquat detection. Reproduced from Barshevskaya et al., 2025 [55]. (**e**) AuNP-based CSA for pesticide discrimination. Reproduced from Zhao et al., 2023 [56]. (**f**) Chloride-regulated triangular AgNP etching for bacterial identification. Reproduced from Liu et al., 2025 [57]. (**g**) Single-atom Co nanozyme array for sulfur-containing metal salt discrimination. Reproduced from Wang et al., 2023 [58]. (**h**) Magnetic bead-assisted paper sensor for foodborne pathogen detection. Reproduced from Hong et al., 2024 [59]. (**i**) MOF-enhanced CSA for early detection of citrus infestation by Bactrocera dorsalis. Reproduced from Zhang et al., 2026 [60].

Organic sensing materials are mainly used to translate local chemical interactions into visible optical changes. Chemoresponsive dyes, including pH indicators, porphyrins, metalloporphyrins and natural pigments, are widely used because of their low cost, easy immobilization and intuitive color responses toward VOCs, acids, bases and amine-related spoilage markers. Natural pigments are particularly attractive for food-related CSA applications due to their biocompatibility and low toxicity, although their stability and batch consistency still require attention. Jiang et al. constructed a natural pigment array from anthocyanin-derived dyes for AFB1-related wheat quality assessment; in this case, the array responded to mold-associated volatile profiles rather than directly recognizing AFB1 molecules (Figure 3a) [52]. Organic nanoparticles and functional polymer matrices can further improve dispersion, dye retention, gas accessibility and multifunctional readout. In another example, flavonol nanocrystals were incorporated into packaging films to preserve banana freshness and provide colorimetric/fluorescent freshness indication (Figure 3b) [53]. In polymer-based systems, polydiacetylene hydrogel beads can undergo a blue-to-red transition after exposure to biogenic amines from spoiled meat, giving a visible signal for meat spoilage (Figure 3c) [54]. For targets requiring higher specificity, immunoassay formats can also be introduced. Barshevskaya et al combined magnetic preconcentration with AuNP-based signal amplification in a lateral flow assay for paraquat detection (Figure 3d) [55]. Overall, dye- and pigment-based arrays are suitable for rapid screening and freshness indication, polymer or hydrogel systems are useful for stable and gas-accessible sensing interfaces, and immunoassay formats are more appropriate for target-specific contaminant detection.

Inorganic materials provide optical, catalytic and enrichment functions that are difficult to achieve with molecular dyes alone. AuNPs and AgNPs are representative plasmonic materials because their aggregation, dispersion or etching can generate clear color changes. AuNP-based arrays are suitable for rapid pesticide screening, as demonstrated by a smartphone-assisted CSA in which pesticide-regulated acetylcholinesterase activity induced AuNP aggregation (Figure 3e) [56]. Liu et al. used chloride-regulated etching of triangular AgNPs for bacterial identification in water samples; different bacterial surfaces inhibited AgNP etching to different extents and generated distinct color patterns (Figure 3f) [57]. Nanozyme-based arrays further introduce catalytic signal amplification. For example, single-atom Co sites embedded in nitrogen-doped graphene were used to catalyze TMB oxidation and generate colorimetric fingerprints for sulfur-containing metal salts (Figure 3g) [58]. MNPs mainly serve as enrichment and separation materials rather than direct chromogenic elements. In the pathogen detection system reported by Hong et al. [59], phage-derived bacterial-binding protein-functionalized magnetic beads captured and enriched target bacteria before enzymatic color development on a smartphone-assisted paper sensor (Figure 3h). Zhang et al. integrated pH-sensitive dyes into ZIF-7 nanoparticles to prepare a MOF-enhanced CSA for early detection of citrus infestation by Bactrocera dorsalis (Figure 3i) [60]. The porous ZIF-7 framework helped stabilize the dyes and enrich citrus-emitted VOCs. Therefore, AuNPs and AgNPs are more suitable for plasmonic visual screening, nanozymes for catalytic amplification, MNPs for target enrichment, and MOFs for VOC enrichment and interface regulation.

In the current CSA design, organic and inorganic components are often combined: organic dyes or biorecognition elements provide chemical responsiveness, while inorganic nanomaterials improve stability, enrichment, signal amplification and image-based readout. Therefore, organic–inorganic hybrid sensing systems are becoming a practical strategy for tailoring high-performance CSA response matrices in food analysis [51]. For example, Lin et al. constructed a composite nano-CSA by integrating metalloporphyrin/pH-indicator complex dyes with ZIF-8- and PSA-modified BODIPY dyes. The optimized array improved VOC response sensitivity and increased stability by 8.7–33.3%. When combined with the SVM model, it achieved classification accuracies of 100% and 95.83% for oolong tea flavor intensity and grade level, respectively [61]. Similarly, Yu et al. fabricated a 3 × 4 nanocomposite CSA using UiO-66-NH2 and UiO-66-NO2 to enhance VOC enrichment and sensor response. The array, combined with chemometric models, enabled black garlic processing-stage discrimination with 100% prediction accuracy using ANN and odor sensory score prediction with an SVR correlation coefficient of 0.8919 [62]. In another taste-oriented design, Yu et al. developed a 3 × 3 multi-channel CSA based on pH-indicator color changes, indicator displacement assay, and AgNPRs. Coupled with SVR models, the array enabled quantitative prediction of reducing sugars, amino acid nitrogen, total acid, and taste sensory attributes of black garlic, with prediction correlation coefficients of 0.9863, 0.9232, 0.9666, and 0.9170, respectively [63]. These studies indicate that hybrid CSA systems can broaden response dimensions and improve applicability to complex food matrices.

## 3. Application of CSA Technology in Food Spoilage Detection

**Figure 4:**
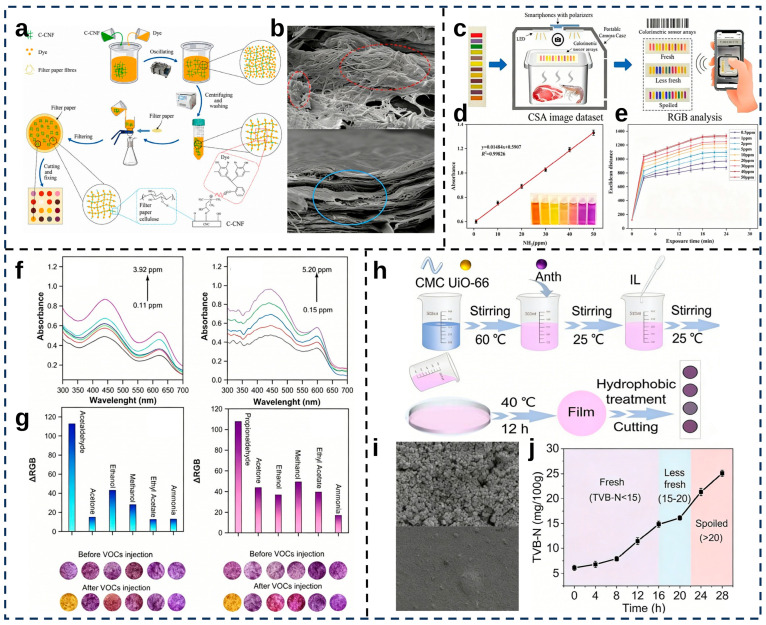
Representative material engineering strategies for CSA-based food spoilage detection. (**a**) Preparation of a C-CNF-mediated paper-based odor sensor array; (**b**) SEM images showing the porous C-CNF dye-anchoring interface. Reproduced from Jiang et al., 2024 [23]. (**c**) Drop-on-demand printed CSA integrated with a portable imaging system for meat freshness assessment; (**d**) NH_3_ calibration curve of the printed CSA; (**e**) time-dependent color-difference responses toward spoilage-related amines. Reproduced from Shi et al., 2025 [65]. (**f**) UV-Vis spectral responses of hydrogel-based freshness indicators toward fruit-derived VOCs; (**g**) selectivity and visible color changes in the hydrogel indicators before and after VOC exposure. Reproduced from Esmaeili et al., 2025 [66]. (**h**) Fabrication process of the CMC/UiO-66/ionic-liquid-tuned anthocyanin film with hydrophobic treatment; (**i**) surface morphology of the film after hydrophobic modification; (**j**) TVB-N evolution during pork storage. Reproduced from Zhao et al., 2025 [21].

Food spoilage is a dynamic biochemical process involving microbial growth, enzymatic degradation, lipid oxidation, and volatile metabolite accumulation. Depending on the food matrix, freshness-related signals may include ammonia, trimethylamine, TVB-N, H_2_S, biogenic amines, aldehydes, organic acids, ethylene, and pH changes [64]. Jiang et al. developed a paper-based odor sensor array inspired by the human olfactory system, in which sixteen chemoresponsive dyes were immobilized on ammonium quaternized cellulose nanofibres (C-CNFs) (Figure 4a). The C-CNF layer provided ionic interactions for dye anchoring and formed a porous network for gas diffusion. SEM images showed that the modified substrate had a more open and layered structure than the original filter paper, which helped reduce dye leakage and improve the accessibility of spoilage-related volatiles, including ammonia, dimethylamine, trimethylamine, cadaverine, and putrescine (Figure 4b) [23]. To reduce variation among sensing spots and batches, Shi et al. used drop-on-demand printing to prepare a spoilage-responsive CSA based on twelve pH-responsive indicators (Figure 4c) [65]. The printed CSA showed a linear response to NH_3_ and clear color-difference changes toward meat-spoilage amines at different exposure times (Figure 4d,e). Its colorimetric fingerprints were further correlated with TVB-N values, supporting the use of printed arrays for reproducible freshness assessment.

Hydrogel matrices have been used to regulate the local sensing environment. Esmaeili et al. reported a hydrogel-based freshness indicator array for fruit-derived VOCs [66], including acetaldehyde, propionaldehyde, acetone, ethanol, methanol, ethyl acetate, and ammonia. UV-Vis spectra showed concentration-dependent responses to acetaldehyde and propionaldehyde (Figure 4f), while selectivity tests and visible color changes confirmed different responses toward representative aldehydes, ketones, alcohols, esters, and ammonia (Figure 4g). For pork freshness monitoring, Zhao et al. prepared a carboxymethyl cellulose-based sensor film containing UiO-66, ionic liquid-tuned anthocyanins, and a hydrophobic nano-silica coating (Figure 4h) [21]. SEM images confirmed the formation of a rough hydrophobic surface after nano-silica modification (Figure 4i), and TVB-N measurements showed the transition from fresh to spoiled pork during storage (Figure 4j). Together, these studies suggest that reliable CSA-based spoilage detection depends on more than dye responsiveness; dye immobilization, gas diffusion, fabrication reproducibility, microenvironment regulation and humidity resistance are also critical.

**Figure 5:**
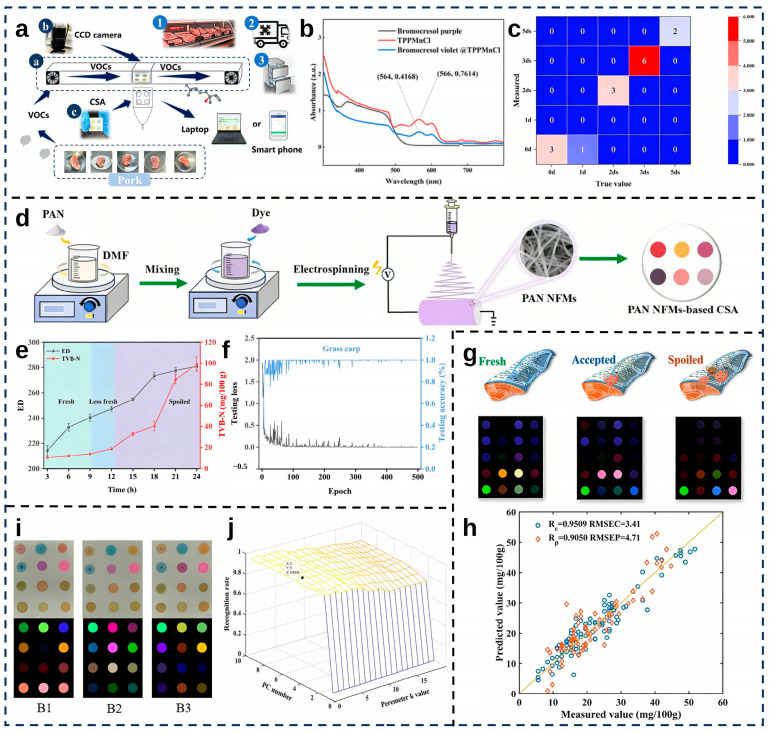
Data analysis strategies for CSA-based food spoilage assessment. (**a**) Pork freshness monitoring workflow based on headspace exposure, CSA imaging, and RGB analysis; (**b**) UV-Vis spectral responses of pH indicator, porphyrin, and mixed dye; (**c**) confusion matrix for pork storage-stage classification. Reproduced from Huang et al., 2025 [67]. (**d**) Preparation of PAN nanofiber mat-based CSA by electrospinning; (**e**) changes in Euclidean distance and TVB-N values during grass carp storage; (**f**) testing loss and accuracy curves of the deep-learning model for grass carp freshness classification. Reproduced from Zhang et al., 2025 [49]. (**g**) CSA color fingerprints of fish samples at fresh, accepted, and spoiled stages; (**h**) regression analysis between predicted and measured TVB-N values. Reproduced from Zhang et al., 2024 [12]. (**i**) CSA images and difference maps for fresh, dry-rot, and soft-rot potato samples; (**j**) KNN parameter optimization and recognition-rate analysis for potato quality classification. Reproduced from Wu et al., 2023 [10].

Once stable colorimetric responses are generated, image acquisition and data processing become necessary to translate visible color changes into freshness-related information. In practical food monitoring, spoilage is a continuous process, and color changes therefore need to be correlated with storage time, freshness grades, or reference indicators such as TVB-N. Huang et al. developed a pork freshness monitoring system based on a porphyrin-pH indicator CSA [67]. The system combined headspace exposure, CCD or smartphone imaging, and RGB analysis to evaluate pork freshness in retail, transportation, and refrigerator storage scenarios (Figure 5a). UV-Vis spectra showed the spectral variation in the selected pH indicator, porphyrin, and mixed dye, supporting the role of dye-porphyrin interactions in stabilizing the sensing units (Figure 5b). The classification results further showed that image-derived features could distinguish different pork storage stages (Figure 5c). For chicken meat freshness prediction, Geng et al. fabricated a nine-dye CSA and compared linear and nonlinear regression models for TVB-N estimation. Among the tested models, CARS-SVM provided the best prediction performance, supporting the use of variable selection and nonlinear calibration for quantitative freshness assessment [68]. For fish freshness assessment, Zhang et al. prepared a PAN nanofiber mat-based CSA by electrospinning, in which the porous nanofiber structure facilitated the diffusion of spoilage-related volatiles (Figure 5d). During storage, the Euclidean distance values extracted from CSA images increased together with TVB-N contents, indicating a correlation between colorimetric response and chemical spoilage indicators (Figure 5e). The testing loss and accuracy curves of the ResNet-based model further showed the feasibility of using CSA images for freshness classification (Figure 5f) [49]. In another study on fish samples, CSA color fingerprints clearly changed across fresh, accepted, and spoiled stages (Figure 5g), and regression analysis showed good agreement between predicted and measured TVB-N values (Figure 5h) [12]. Pattern recognition methods have also been used to convert CSA responses into direct quality categories. Wu et al. applied a visible CSA combined with chemometric algorithms for potato quality monitoring. CSA images and difference maps provided distinguishable colorimetric patterns for fresh, dry-rot and soft-rot potato samples (Figure 5i). By optimizing the number of principal components and the K value in the KNN model, the recognition rate was evaluated for potato classification (Figure 5j) [10]. Similarly, Liang et al. coupled CSA with machine learning approaches to discriminate the freshness of grain matrices, including rice, paddy, and soybean. In this work, VOC-induced CSA responses were analyzed by image processing and Vis-NIR spectral acquisition, and LDA/KNN models were used to classify grains with different freshness degrees [69]. Overall, image acquisition, color feature extraction, correlation with reference indicators, and pattern recognition help translate CSA responses into more reproducible spoilage assessment results.

Overall, the reported CSA systems for food spoilage detection show different strengths and limitations depending on sensing materials, fabrication methods, readout strategies and target food matrices. Dye-based and natural-pigment-based arrays are simple, low-cost and suitable for rapid visual freshness screening, especially when spoilage is accompanied by pH changes, volatile amines or other headspace metabolites. However, their practical performance may be affected by dye stability, humidity, illumination conditions and batch-to-batch variation. Printed arrays and polymer/hydrogel-supported CSAs improve fabrication reproducibility, dye immobilization and gas accessibility, but their response rate and sensitivity may still depend on substrate structure, swelling behavior and volatile diffusion. Nanofiber- or MOF-assisted arrays can enhance analyte enrichment and sensing-interface stability, whereas their preparation complexity and long-term storage stability should be further evaluated. From the perspective of practical food monitoring, smartphone- or camera-assisted CSAs are more suitable for on-site screening and intelligent packaging, while systems combined with multispectral imaging, deep learning or complex chemometric models may provide higher accuracy but require more standardized imaging conditions, larger datasets and external validation. Therefore, future CSA-based spoilage detection should balance sensitivity, reproducibility, portability, matrix tolerance and model robustness according to specific food products and monitoring scenarios.

## 4. Application of CSA Technology in Food Adulteration Detection

Following freshness and spoilage assessment, another important application of CSA technology is the identification of food adulteration. Food spoilage detection usually focuses on dynamic biochemical changes caused by microbial growth, enzymatic degradation or volatile metabolite accumulation during storage. In contrast, food adulteration detection aims to identify intentional or unintentional substitution, dilution or mislabeling, which often produces subtler differences in composition, aroma or optical response rather than continuous deterioration signals. Therefore, CSA-based adulteration analysis requires discriminating characteristic fingerprints associated with authenticity, origin or compositional integrity.

**Figure 6:**
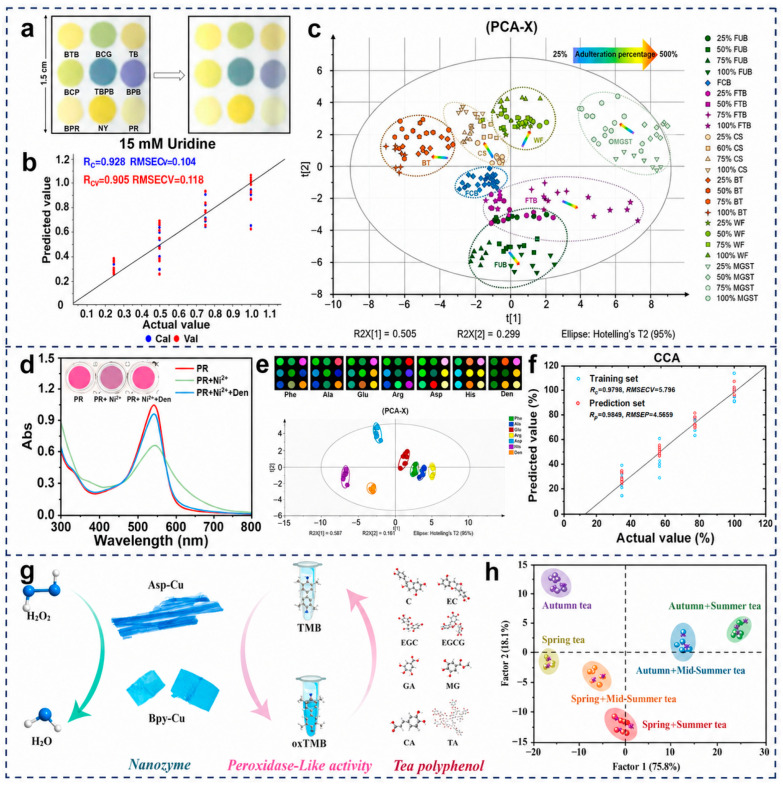
Liquid phase CSA fingerprinting for food adulteration identification. (**a**) Inkjet-printed pH-indicator CSA before and after exposure to 15 mM uridine for evaluating the response toward Fritillariae cirrhosae bulbus-related 1,2-diol/catechol-type constituents; (**b**) regression analysis between actual and predicted adulteration values based on the PLS model; (**c**) PCA score plot for distinguishing pure FCB and six adulterants at different adulteration percentages. Reproduced from Li et al., 2022 [70]. (**d**) UV-Vis spectral and visual color changes in the PR/Ni^2+^ indicator displacement system after interaction with dencichine; (**e**) colorimetric fingerprints and PCA of amino acids and PN-related characteristic constituents; (**f**) correlation between actual and predicted adulteration values for CCA-adulterated PN samples based on the SVR model. Reproduced from Li et al., 2024 [71]. (**g**) Copper-based nanozyme sensing mechanism involving peroxidase-like TMB oxidation and inhibition by tea polyphenols; (**h**) score plot for discriminating Tieguanyin tea samples with different seasonal adulteration patterns. Reproduced from Yang et al., 2024 [72].

In liquid-phase CSA fingerprinting, sample extracts are directly analyzed to reveal compositional differences associated with adulteration. For the authentication of Fritillariae cirrhosae bulbus and its adulterants, Li et al. fabricated an inkjet-printed pH-indicator CSA on a mixed cellulose ester membrane [70]. The array showed obvious color changes after exposure to uridine, indicating its response to FCB-related 1,2-diol/catechol-type constituents (Figure 6a). The quantitative model showed good agreement between actual and predicted adulteration values (Figure 6b), while PCA further distinguished pure FCB from six adulterants at different adulteration percentages (Figure 6c). For adulterated Panax notoginseng powder, Li et al. constructed a PR/Ni^2+^-based indicator displacement array [71]. The addition of dencichine induced UV-Vis spectral and visible color changes, supporting the competitive coordination response mechanism (Figure 6d). The colorimetric fingerprints and PCA results showed that the array could distinguish amino acids and PN-related constituents, and SVR regression further enabled prediction of CCA adulteration levels (Figure 6e,f). In the Tieguanyin adulteration analysis, Yang et al. introduced Bpy-Cu and Asp-Cu nanozymes with peroxidase-like activity [72]. Tea polyphenols inhibited nanozyme-catalyzed TMB oxidation to different extents, generating catalytic colorimetric fingerprints for tea polyphenol recognition and seasonal adulteration discrimination (Figure 6g,h).

**Figure 7:**
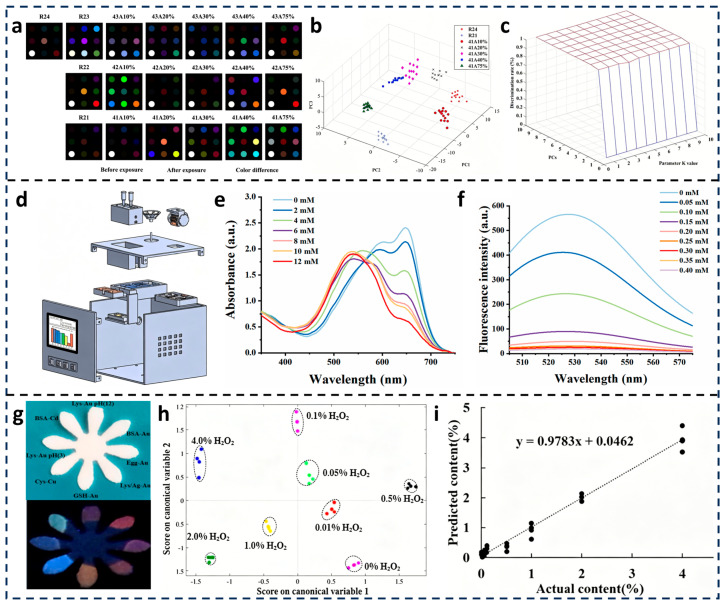
Headspace and multimodal sensing strategies for CSA-based food adulteration identification. (**a**) CSA images before exposure, after exposure, and corresponding color-difference maps for rice adulteration analysis; (**b**) three-dimensional PCA score plot for distinguishing freshly harvested, stored, and adulterated rice samples; (**c**) recognition-rate analysis based on optimized kNN parameters. Reproduced from Arslan et al., 2025 [74]. (**d**) Portable LAMP device integrating colorimetric and fluorescence dual-channel detection for meat adulteration identification; (**e**) UV-Vis absorbance responses of the colorimetric channel; (**f**) fluorescence intensity responses of the fluorescence channel. Reproduced from Fang et al., 2025 [77]. (**g**) Paper-based fluorescent optical tongue array under visible and UV illumination for milk adulterant detection; (**h**) canonical variate analysis for discriminating different H_2_O_2_ adulteration levels. (**i**) Regression analysis between actual and predicted H_2_O_2_ contents. Reproduced from Ghohestani et al., 2024 [78].

Arslan et al. developed a rapid, minimally destructive headspace sensing strategy for rice adulteration by constructing a smart olfactory visualization system. Earlier, they built a smartphone-assisted CSA on a PVDF membrane and used PCA, HCA, and kNN to analyze volatile colorimetric fingerprints for basmati rice adulteration detection [73]. Freshly harvested R24 rice was mixed with stored R23, R22, and R21 rice at different adulteration levels. The CSA generated distinct before-exposure, after-exposure, and color-difference maps for different rice samples (Figure 7a). PCA separated freshly harvested, stored, and adulterated rice samples in a three-dimensional score space (Figure 7b), and the optimized kNN model further verified the classification performance (Figure 7c) [74]. A similar headspace strategy was reported for peanut seed oil adulterated with rapeseed oil, where CSA-derived volatile fingerprints were combined with PCA, HCA, and kNN models for rapid discrimination of different adulteration levels [24]. For solid and semi-solid matrices, Liu et al. designed a Dyes/Dyes-Cu-MOF paper-based CSA for mildewed wheat detection [75]. The array captured volatile gases released from wheat with different mildew rates, and the extracted ΔRGB values were further processed by pattern recognition, with LDA achieving 100% discrimination of all samples. Han et al. developed a low-cost electronic nose based on colorimetric sensors for detecting beef adulterated with pork [76]. In this work, colorimetric responses to meat-derived VOCs were modeled using Fisher LDA, ELM, and BP-ANN, enabling both qualitative identification of beef–pork mixtures and quantitative prediction of adulteration levels.

CSA-based adulteration analysis can be further enhanced by integrating colorimetric responses with fluorescence or Vis-NIR spectral information. Fang et al. developed a portable dual-channel platform for on-site meat adulteration identification [77], in which hydroxynaphthol blue colorimetry and calcein fluorescence were integrated into one device (Figure 7d). The absorbance and fluorescence spectra verified the feasibility of the two optical readout channels, providing complementary visual and fluorescent evidence for pork adulteration detection (Figure 7e,f). Ghohestani et al. constructed a paper-based fluorescent optical tongue using eight nanoclusters for milk source identification and adulterant quantification [78]. The flower-shaped array produced distinguishable visible and fluorescent responses (Figure 7g), while canonical variate analysis separated different H_2_O_2_ adulteration levels (Figure 7h), and PLS regression enabled quantitative prediction of H_2_O_2_ content (Figure 7i). In Vis-NIR-assisted systems, Ouyang et al. combined CSA with visible near-infrared spectroscopy for matcha classification, where the reacted color-sensitive dyes provided spectral information and the BPANN model achieved high classification accuracy [20]. Zhang et al. further developed an anti-interference CSA coupled with near-infrared spectroscopy for qualitative and quantitative detection of Pb and Hg in corn oil, showing that Vis-NIR information can compensate for the limited dimensionality of RGB-based CSA features [79].

Overall, the suitability of CSA-based food adulteration detection depends largely on the physical form of the food matrix, the dominant chemical information available, and the intended analytical objective. Liquid-phase CSA fingerprinting is particularly suitable for powdered or extractable samples because it can capture non-volatile compositional differences and support quantitative prediction of adulteration levels. However, its reproducibility may be affected by extraction conditions, solvent effects, and matrix interference. In contrast, headspace CSA requires less sample pretreatment and enables rapid and minimally destructive screening of foods with informative volatile profiles, such as grains, oils, meats, and tea, although its performance is more sensitive to temperature, humidity, storage history, and headspace-equilibration conditions. When conventional colorimetric fingerprints provide insufficient discriminatory information, complementary fluorescence, Vis-NIR spectral, or gas-sensing signals can be introduced to expand data dimensions and improve discrimination ability. Such multimodal approaches may enhance analytical specificity and reliability, but they can also increase hardware complexity, calibration requirements, reaction steps, and data-processing burden. Feature-level fusion between CSA and electronic-nose arrays provides a representative example of this strategy [80]. Compared with spoilage detection, adulteration detection often requires the recognition of subtle and sometimes non-progressive compositional differences; therefore, representative calibration sets, matrix-interference control, robust feature extraction, and model transferability are particularly important. Future studies should focus on establishing matrix- and adulterant-specific colorimetric fingerprint databases, improving the quantitative identification of low-level and mixed adulteration, and developing low-cost paper-based devices or simplified multimodal CSA platforms for the rapid screening of specific adulterants in commonly adulterated foods.

## 5. Application of CSA Technology in Food Composition and Contamination Detection

**Figure 8:**
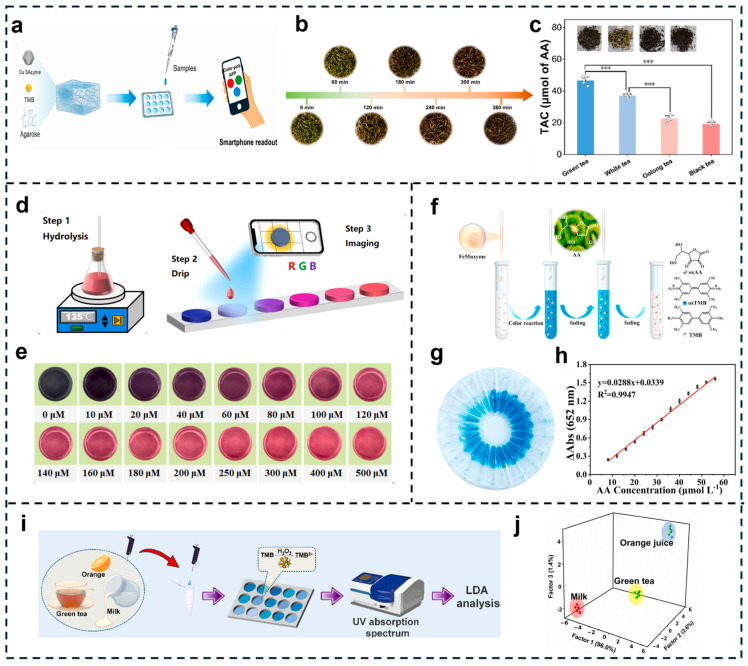
Applications of colorimetric sensor arrays in food quality assessment. (**a**) Schematic of the dual-mode liquid/gel sensor system; (**b**) photographs of tea leaves at different fermentation times, showing color changes during fermentation; (**c**) dynamic variation in TAC during black tea fermentation, measured by the nanozyme platform. *** indicate a significant difference (*p* < 0.001) from the corresponding control group. Reproduced from Shen et al., 2025 [84]. (**d**) Schematic illustration of the smartphone-assisted hydrogel sensor design; (**e**) colorimetric response and quantitative analysis of glucose (converted from starch) through H_2_O_2_-mediated etching; Reproduced from Xu et al., 2025 [85]. (**f**) Schematic of the FeMnzyme/oxTMB colorimetric sensor; (**g**) concentration-dependent color changes in the sensor upon AA addition; (**h**) selectivity analysis showing minimal interference from other common substances. Reproduced from Han et al., 2022 [86]. (**i**) Schematic of the sensor array for TAC identification across different foods; (**j**) TAC “fingerprints” of milk, green tea and orange juice, demonstrating differential antioxidant levels and their potential for overall quality assessment. Reproduced from Wu et al., 2023 [87].

Colorimetric sensor array-based analysis of endogenous food components covers functional active substances and characteristic chemical markers in a variety of food matrices [81,82,83], providing a rapid and visual analytical means for food quality evaluation. For example, Shen et al. constructed a Cu single-atom nanozyme platform to evaluate the total antioxidant capacity (TAC) of fruit juices and tea via the oxidation of 3,3′,5,5′-tetramethylbenzidine (TMB) (Figure 8a) [84]. During tea fermentation, the TAC variation corresponded to changes in leaf color, which gradually transitioned from light green to brown over time, and the dynamic TAC curve enabled quantitative monitoring of the antioxidant activity throughout the fermentation process (Figure 8b,c). Meanwhile, Xu et al. developed an Au@MnO_2_ hydrogel sensor for the detection of starch in fermented grains (Figure 8d) [85]. After acid hydrolysis converted starch to glucose, glucose oxidase (GOx) catalyzed its oxidation to generate H_2_O_2_, which etched the nanoparticles and triggered a colorimetric response in the hydrogel (Figure 8e). The color changes were captured using a smartphone and analyzed via the RGB channels to quantify glucose concentration and subsequently determine starch content. Furthermore, Han et al. employed a Fe-Mn bimetallic nanozyme to quantify ascorbic acid (AA) in fruits and vegetables (Figure 8f) [86]. The Fe-Mn nanozyme catalyzed the oxidation of TMB to produce a blue product in the absence of exogenous H_2_O_2_, with adsorbed oxygen generating superoxide radicals to drive the reaction. Increasing AA concentrations gradually converted the blue product into a colorless form, producing a concentration-dependent colorimetric response (Figure 8g), and monitoring the absorbance enabled quantification of AA levels (Figure 8h). In addition, Wu et al. constructed an Au_2_Pt three-channel nanozyme sensor array capable of distinguishing multiple antioxidants in milk and juice samples [87]. Different antioxidants reduced TMB to varying degrees, generating distinct colorimetric responses in each sensor unit. Recording these absorbance changes produced optical fingerprints of the antioxidants (Figure 8i), which were further analyzed using linear discriminant analysis (LDA) to differentiate the TAC of different food samples (Figure 8j). Collectively, these studies illustrate the diverse applications of colorimetric sensor arrays in food quality assessment, including visual monitoring, quantitative analysis, and multidimensional profiling. In the representative studies reviewed here, endogenous food components generally occur at higher concentrations than trace contaminants. Accordingly, these platforms mainly emphasize the establishment of concentration-dependent color responses and the accuracy of quantitative calibration. Their analytical performance, therefore, depends strongly on reaction linearity, signal reproducibility, and the consistency between colorimetric outputs and reference measurements.

**Figure 9:**
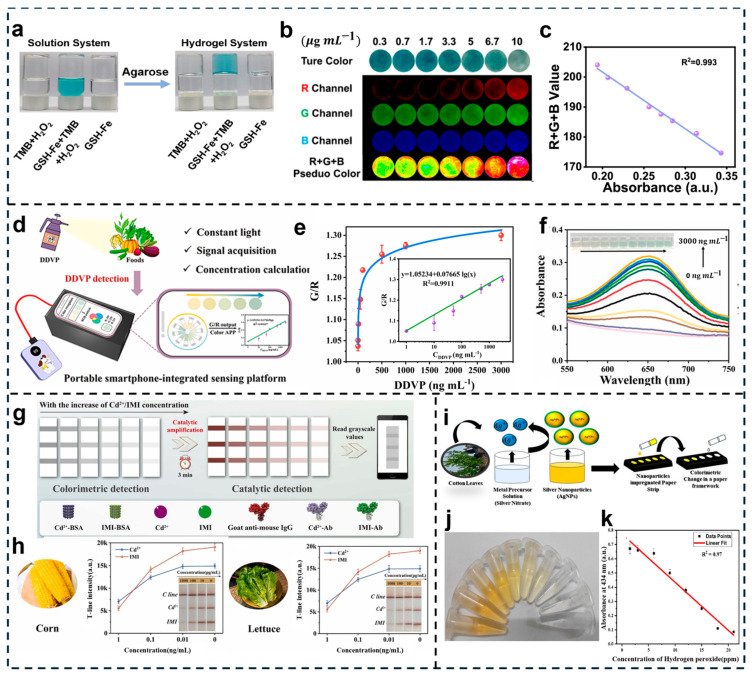
Application of colorimetric sensor arrays in food chemical contamination detection. (**a**) Photograph and schematic of the liquid/gel dual-mode sensing system; (**b**) true-color images split into RGB channels with (R+G+B) values calculated to generate pseudo-color images for quantitative analysis. In the pseudo-color images, the red signal intensity increases with increasing thiram concentration, facilitating rapid visual assessment; (**c**) comparison of image-processing results with commercial microplate reader absorbance measurements and thermal stability analysis of the hydrogel. Reproduced from Yan et al., 2024 [25]. (**d**) Design schematic of the FeMnOx nanozyme-based portable smartphone platform; (**e**) absorbance variation and linear correlation at different DDVP concentrations; (**f**) absorption spectra at 0–3000 ng/mL DDVP and corresponding photographs. Reproduced from Liu et al., 2024 [91]. (**g**) Schematic illustration of standard colorimetric mode and catalytic signal enhancement mode; (**h**) T-line color changes and grayscale analysis for corn and lettuce samples, used for quantitative evaluation of analyte concentration. Reproduced from Zhang et al., 2025 [92]. (**i**) Schematic of the AgNPs sensor construction and detection principle on liquid and paper-based platforms; (**j**) colorimetric changes in milk spiked with different H_2_O_2_ concentrations; (**k**) calibration curve of absorbance versus H_2_O_2_ concentration for quantitative analysis. Reproduced from Das et al., 2024 [93].

In contrast to endogenous component analysis, food-contaminant detection generally targets hazardous analytes present at relatively low concentrations in complex food matrices. Accordingly, these applications place greater emphasis on selective recognition, interference suppression, and signal amplification, while quantitative accuracy and recovery in real samples remain essential. Colorimetric sensor arrays can effectively detect pollutants such as pesticide residues [88], heavy metal ions [89], and mycotoxins [90] in various food matrices, demonstrating good applicability for rapid detection of real samples. For example, Xu et al. developed a glutathione–iron (GSH-Fe) nanozyme hydrogel system for the detection of the pesticide thiram [25]. Within an agarose hydrogel matrix, this system catalyzed the oxidation of TMB by H_2_O_2_, generating a distinct color change from deep blue to light blue, which could be quantitatively analyzed via RGB image analysis (Figure 9a,b). The nanozyme maintained stable catalytic activity across different temperatures, and no significant activity loss was observed after 30 days of storage. The hydrogel platform exhibited a detection limit of 0.3 μg/mL (Figure 9c). In another study, Liu et al. employed a Fe-Mn bimetallic oxide (FeMnOx) nanozyme to construct a portable smartphone-based platform for the detection of DDVP (Figure 9d) [91]. In this system, the FeMnOx nanozyme catalyzed the oxidation of TMB to produce a blue product, while acetylcholinesterase (AChE) and its substrate promoted decolorization of the blue product. DDVP inhibited AChE activity, preventing decolorization and thereby generating a concentration-dependent color recovery. The absorbance exhibited a good linear relationship with DDVP concentration over the range of 1–3000 ng/mL (Figure 9e), and the intensity of the UV–Vis absorption peak increased synchronously with concentration (Figure 9f). For the multiplexed detection of contaminants, Zhang et al. developed a paper-based immunochromatographic system based on GO-Pt_30_-AuIr nanozymes [92], capable of simultaneously detecting Cd^2+^ and imidacloprid (IMI) (Figure 9g). In the competitive immunoassay, the color intensity of the T-line decreased with increasing analyte concentration. The catalytic enhancement provided by the nanozyme amplified the color change, improving detection sensitivity. Grayscale analysis revealed a good linear correlation between T-line color intensity and the concentrations of Cd^2+^ and IMI, with detection limits reaching the pg/mL level in corn and lettuce samples (Figure 9h). Additionally, Das et al. employed green-synthesized silver nanoparticles (AgNPs) to construct a paper-based sensor for H_2_O_2_ detection in milk (Figure 9i) [93]. In a liquid system, H_2_O_2_ oxidized AgNPs, causing a gradual color change from pale yellow to colorless, accompanied by a decrease in absorbance (Figure 9j). Based on this principle, a paper-based detection platform was further developed, which provided more uniform and rapid color responses for pretreated milk samples (Figure 9k). Calibration curve analysis indicated a detection limit of 8.46 ppm, and good selectivity was maintained even in the presence of coexisting interfering substances. Collectively, colorimetric sensor arrays combined with nanozymes or paper-based platforms enable the rapid, visual, and highly sensitive detection of pesticides, heavy metals, and other chemical contaminants. Compared with endogenous component analysis, these systems generally require lower detection limits, stronger resistance to matrix interference, and more selective reaction mechanisms, such as enzyme inhibition, immunorecognition, nanoparticle etching, or catalytic amplification. Endogenous-component platforms often place greater emphasis on quantitative accuracy, calibration range, and response reproducibility, whereas contaminant-detection platforms typically impose additional requirements for sensitivity, selectivity, sample pretreatment, and recovery in real food matrices. However, both types of applications require reliable calibration, reproducible responses, and validation against appropriate reference methods. This distinction indicates that the design of CSA systems should be guided not only by the target concentration but also by analyte origin, matrix complexity, and the required level of analytical reliability.

## 6. Application of CSA Technology in Food Storage and Volatile Compound Detection

**Figure 10:**
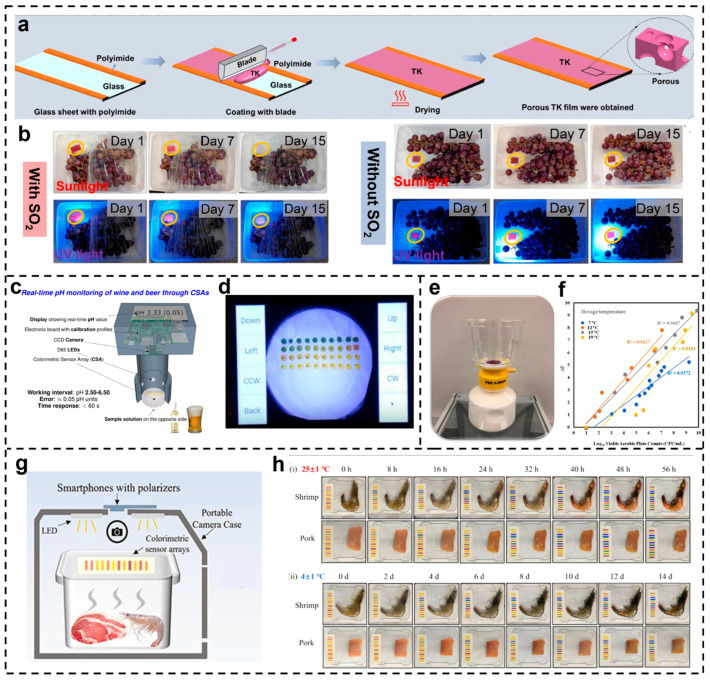
Application of colorimetric sensor arrays in monitoring food storage. (**a**) Schematic of label preparation; (**b**) color changes in the label over storage time under SO_2_ conditions and fluorescence changes under SO_2_-free conditions. Reproduced from Wang et al., 2024 [97]. (**c**) Design and detection principle of the CSA device; (**d**) color changes in white wine, Prosecco, and beer samples during storage and corresponding pH calculation results. Reproduced from Pastore et al., 2024 [98]. (**e**) Sensor appearance and preparation schematic; (**f**) color changes in the sensor during milk storage at different temperatures and the corresponding relationship with aerobic bacterial counts for storage status evaluation. Reproduced from Ziyaina et al., 2019 [99]. (**g**) Color response of the CSA to NH_3_, DMA, and TMA; (**h**) Real-time images and corresponding CSA images of shrimp and pork at different freshness levels, collected at 25 ± 1 °C (i) and 4 ± 1 °C (ii). Reproduced from Shi et al., 2025 [65].

Among the various food detection applications of colorimetric sensor arrays, food storage monitoring represents another important direction, covering grain mildew prevention [94], fresh food preservation [95], and quality change monitoring [96]. For example, Wang et al. developed an SO_2_-responsive smart label (3-TSL-PS) based on tetraketone (TK) derivatives for real-time monitoring of fruits and vegetables during storage [97]. SO_2_ selectively reacts with the C=C double bonds of TK molecules via a Michael addition, disrupting the intramolecular charge transfer effect and inducing a blue shift in the absorption and emission spectra. This results in a visible color change from deep red to pale pink and a fluorescence shift from red to blue (Figure 10a). Under SO_2_-enriched storage conditions, the label’s color difference (ΔE) decreased from 41 to 12, corresponding to a fruit decay rate below 7%, whereas in the SO_2_-free control group, ΔE remained stable at 38–40, with evident surface mold and softening (Figure 10b). The entire detection can be completed within 12 min, providing a simple, rapid, and visual quantification method without complex instrumentation. For beverage storage, Pastore et al. developed a portable CSA device capable of real-time pH monitoring in white wine, Prosecco, and beer [98]. The device derives pH values by combining color changes in a sensing membrane with image processing algorithms, yielding results highly consistent with conventional glass electrodes, with deviations of only 0.01–0.05 pH units and stable readings without frequent recalibration (Figure 10c). During a 10-day semi-open storage of white wine, the CSA continuously captured the gradual pH decrease, indirectly reflecting increased microbial metabolic activity (Figure 10d). Additionally, Ziyaina et al. constructed a Schiff reagent-functionalized SiO_2_ nanoparticle paper-based sensor for real-time monitoring of dairy products [99]. The sensor selectively captures aldehyde and ketone spoilage markers in the headspace, producing a color change from pink to purple that is quantified as ΔE (Figure 10e). ΔE values increase linearly with aerobic bacterial counts at different storage temperatures, with R^2^ values ranging from 0.81 to 0.96, and temperature significantly affects the regression slopes (*p* < 0.05). Based on ΔE, dairy products can be classified into freshness levels: ΔE below 3 indicates fresh, 6–9 indicates questionable, and above 9 indicates nearing the end of shelf life, with all measurements performed in a non-contact headspace mode (Figure 10f). For meat cold-chain monitoring, Shi et al. developed a 12-channel pH-responsive colorimetric sensor array for pork and shrimp [65]. The array detects ammonia, dimethylamine, and trimethylamine at concentrations of 5–50 mg/L and is exposed to the headspace of cold-chain samples in a non-contact manner (Figure 10g). The total color difference varies over storage time and exhibits a strong linear correlation with total volatile basic nitrogen (TVB-N) measurements (R^2^ = 0.92) (Figure 10h). By applying Euclidean distance and pattern analysis, the array enables clear clustering of samples according to storage duration and freshness level, supporting continuous, non-contact monitoring of meat freshness.

**Figure 11:**
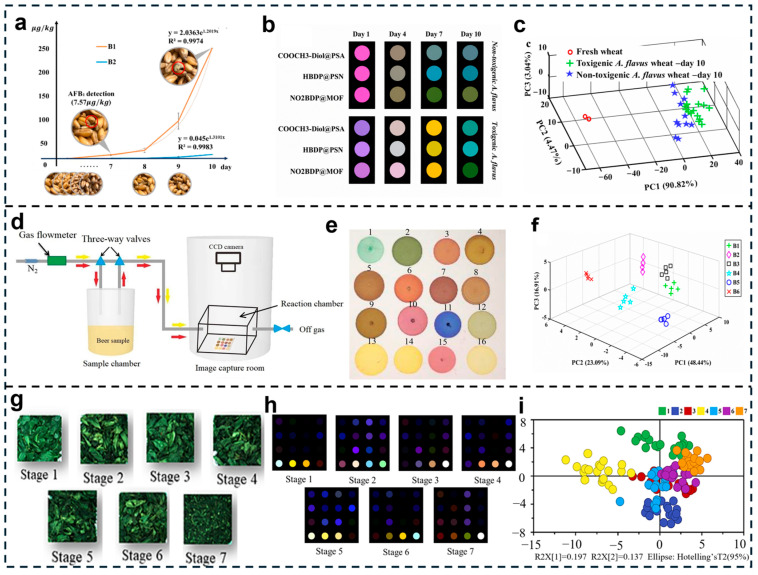
Colorimetric sensor array applications for volatile compound detection. (**a**) Line graph of AFB1 and AFB2 levels and grain characteristics of wheat infected with toxigenic *A. flavus* over time, with arrows indicating the corresponding infection stages; (**b**) RGB difference map showing color responses at selected sensor spots for differentiating toxigenic, non-toxigenic, and fresh wheat, where the colors indicate RGB differences before and after exposure to wheat VOCs; (**c**) PCA model based on color responses displaying clustering of the three sample types in principal component space, with different colors/symbols representing different wheat sample groups. Reproduced from Chen et al., 2024 [103]. (**d**) Schematic of the CSA and dedicated reaction device for capturing beer volatiles, with arrows indicating the volatile-transfer and detection workflow; (**e**) CSA images showing color response patterns across sensor spots for six beer samples, where the spot colors represent the RGB response patterns of different chemically responsive dyes after exposure to beer volatiles; (**f**) three-dimensional PCA score plot constructed from GC-MS data showing clustering of the six beer samples, with different colors/symbols representing different beer types. Reproduced from Yang et al., 2021 [104]. (**g**) Representative tencha samples collected from seven drying stages, corresponding to the sam-ple-display step in the original olfactory visualization sensor-system procedure; (**h**) CSA images showing color intensity and distribution differences at different drying stages, where the colors indicate RGB response differences of the CSA to volatile aroma compounds re-leased during tencha drying; (**i**) OPLS-DA model based on CSA images differentiating seven drying stages, with different colors representing different drying-stage groups. Reproduced from Liu et al., 2024 [105].

Whereas the storage-monitoring studies discussed above emphasize longitudinal tracking of quality changes, the VOC-fingerprinting studies reviewed in the following section generally classify sample identity or quality state at defined sampling points. Colorimetric sensor arrays based on volatile substance detection have been extensively studied, covering the analysis of characteristic volatiles in a variety of food matrices [100,101,102]. This technique shows good applicability for the rapid detection of real samples. In the representative VOC-fingerprinting studies reviewed here, the key requirement is not necessarily long-term signal tracking, but the reproducible capture of multichannel headspace response patterns under standardized sampling conditions. For example, Chen et al. [103] employed a nanocomposite-modified CSA to differentiate wheat infected with toxigenic and non-toxigenic Aspergillus flavus (Figure 11a). The sensor array generated differential RGB responses to three key VOCs (2-methylbutanal, hexanal, and 1-pentanol) while showing weak responses to interfering compounds, enabling clear discrimination among toxigenic, non-toxigenic, and fresh wheat samples (Figure 11b). Principal component analysis (PCA) of the RGB responses showed that the first three principal components cumulatively explained 99.32% of the variance, and linear discriminant analysis (LDA) based on the first five principal components achieved a classification accuracy of 92.86% (Figure 11c), allowing discrimination between toxigenic and non-toxigenic wheat at different infection stages without the need for labels or complex sample preparation. Furthermore, Yang et al. applied CSA to the analysis of volatile compounds in six beer types [104]. The sensor array was exposed to beer headspace gases via an airstream, inducing color changes in the sensing dyes (Figure 11d). Different beer samples produced differential RGB color responses across multiple sensing spots, enabling visual discrimination of the six beer types (Figure 11e). By extracting the color responses and identifying key VOCs (ethyl caprylate, phenethyl acetate, isoamyl alcohol, and caprylic acid) using GC-MS, a PCA model was constructed that clearly separated the six beer types, with the first three principal components cumulatively explaining 78.29% of the variance (Figure 11f). LDA based on the first three principal components achieved 100% classification accuracy, allowing accurate discrimination of the six beer types without the need for labels. In tea processing monitoring, Liu et al. utilized CSA to analyze VOCs during the drying of Tiancha tea [105]. The sensor array was positioned above the samples to capture VOCs at different drying stages, producing differential RGB color responses (Figure 11g) that reflected dynamic changes in alcohols, aldehydes, and esters (Figure 11h). Based on these color features, an OPLS-DA model clearly separated samples from seven drying stages in the principal component space, with R^2^Y = 0.998 and Q^2^ = 0.988, and random forest prediction achieved an accuracy of 91.07% (Figure 11i), enabling discrimination of tea samples at different drying stages without labels or complex sample preparation. Collectively, these studies illustrate that CSA, combined with pattern recognition algorithms, can achieve rapid and non-destructive assessment of food quality and processing states across diverse food matrices through the capture of headspace VOCs.

From a temporal perspective, storage-monitoring applications require sensors to preserve response stability and calibration consistency during prolonged or repeated measurements, because the objective is to reveal the trajectory of food deterioration and identify the point at which a freshness threshold is exceeded. VOC-fingerprinting applications, by contrast, mainly perform cross-sectional classification at selected sampling stages and therefore depend more strongly on standardized headspace collection, sampling time, temperature, and the spatial uniformity of VOC distribution. The former is suited to continuous shelf-life and cold-chain monitoring, whereas the latter is more appropriate for identifying food varieties, microbial states, or processing stages. Thus, temporal continuity and sampling reproducibility represent two distinct design priorities for CSA applications in food storage and volatile analysis.

## 7. System Integration and Intelligent Detection Platform of CSA Technology

**Figure 12:**
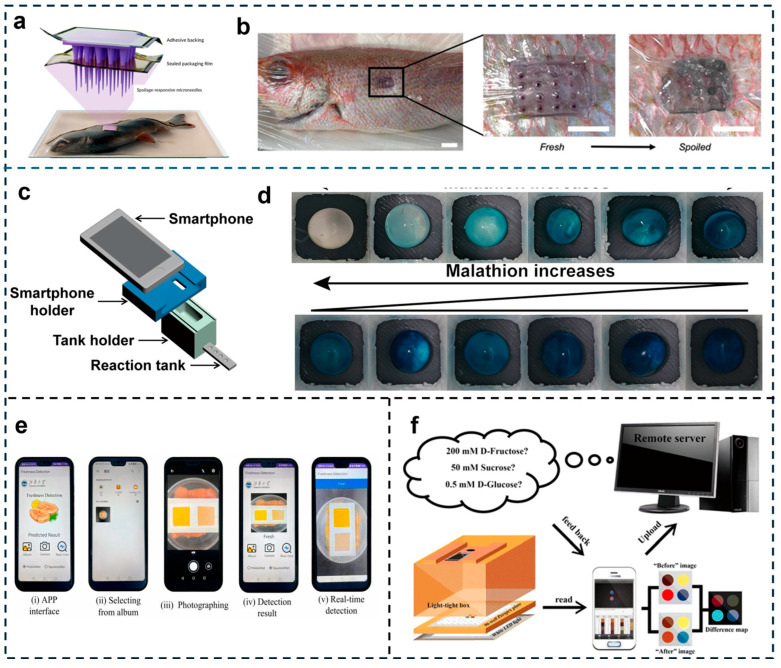
Application of colorimetric sensor arrays in system integration and intelligent detection platforms. (**a**) Line graph showing microneedle color changes and ΔE values over storage time; (**b**) comparison of fish surface conditions and microneedle colors under different storage conditions. Reproduced from Khan et al., 2026 [107]. (**c**) Portable smartphone-assisted sensing device for malathion detection; (**d**) colorimetric responses of the nanozyme hydrogel to increasing malathion concentrations, with the blue color gradually fading as the malathion concentration increases. Reproduced from Liu et al., 2021 [108]. (**e**) Smartphone application workflow for real-time salmon freshness assessment. Reproduced from Wu et al., 2025 [109]. (**f**) Small-scale CSA coupled with a remote server system for sugar-containing beverage detection. Reproduced from Cui et al., 2018 [110].

In recent years, the integration of CSA technology with AI, IoTs and smart portable devices has attracted considerable attention [106], and CSA systems are gradually evolving from traditional laboratory-based analytical tools into integrated and intelligent detection platforms. In smart packaging, Khan et al. developed a gelatin–anthocyanin microneedle sensor for real-time monitoring of fish spoilage in packaged fish (Figure 12a) [107]. The microneedles penetrate the packaging membrane to access the headspace, and anthocyanin responds to pH changes, causing a color shift from purple to blue. The average red channel intensity decreased continuously as spoilage metabolites accumulated, and ΔE values quantitatively reflected the spoilage process (Figure 12b). Integrated into the packaging, the sensor enables continuous monitoring of fish freshness. In portable detection, Liu et al. constructed a chromogenic Ag_3_PO_4_/UiO-66 nanozyme hydrogel for on-site malathion analysis (Figure 12c) [108]. Malathion inhibits the peroxidase-like activity of the nanozyme, causing the hydrogel color to lighten gradually with increasing concentration. ΔE values were linearly correlated with malathion concentration (R^2^ ≥ 0.983), with a detection limit of 7.5 ng/mL (Figure 12d). Coupled with a smartphone imaging platform, the system enables rapid on-site screening of malathion.

In mobile-based intelligent assessment, Wu et al. developed a dual pH indicator membrane (curcumin and alizarin) for salmon freshness monitoring (Figure 12e) [109]. The membrane responds to pH changes and volatile basic nitrogen compounds, producing color changes as spoilage progresses, with ΔE values highly correlated with TVB-N (R^2^ = 0.98). A lightweight MobileNetV2 convolutional neural network trained on these color features was deployed on a smartphone app, enabling real-time image acquisition, processing, and freshness prediction on mobile devices. In IoT-based remote monitoring, Cui et al. developed a 2 × 2 pH indicator CSA coupled with smartphones and a remote server for sugar detection in beverages (Figure 12f) [110]. Sensor images captured by the smartphone were uploaded to the cloud for chemometric analysis, and results were returned to the terminal. The server dynamically updates the database and models, supporting end-to-end remote and automated monitoring.

Collectively, these studies demonstrate that CSA, when integrated with smart packaging, portable devices, mobile computing, and IoT platforms, can form a distributed sensing framework encompassing data acquisition, feature extraction, model inference, and feedback, thereby enabling real-time and intelligent monitoring of food quality and safety. Although these integrated systems show strong application potential, their translation from laboratory prototypes to real-world deployment remains challenging across three interconnected levels. At the sensor level, the primary limitation is stability. Dye leaching, indicator degradation, and humidity-induced drift may still occur during storage and operation. Recent studies based on dye-anchoring matrices and hydrophobic nano-silica coatings suggest that such limitations can be mitigated, although not completely eliminated, through improved interfacial design and immobilization strategies [21,23]. However, material optimization alone is insufficient for practical deployment. At the measurement level, variations in array fabrication, headspace exposure time, imaging devices, optical geometry, and illumination conditions introduce substantial inconsistencies in recorded color responses across platforms. Standardized printing protocols enhance array reproducibility [65], while controlled imaging and reference-based color calibration are essential to reduce device- and environment-dependent variability [111,112]. At the application level, such measurement inconsistencies can propagate into reduced model robustness and limited cross-device generalization. A recent study improved classification performance by incorporating images acquired under multiple lighting conditions and using smartphones from different manufacturers during training [113]. These findings indicate that model robustness can be improved through greater data diversity and experimental standardization. Beyond model performance, large-scale deployment requires scalable and reproducible array fabrication, rigorous batch quality control, compatibility with existing food-packaging and processing systems, acceptable unit costs, and simple operation and maintenance across production and distribution networks. Overall, these challenges suggest that the practical deployment of CSA systems requires a coordinated, layered framework integrating material-level stabilization, measurement standardization, data-level diversification, and deployment-level scalability. Nevertheless, the robustness of such approaches under complex supply-chain environments, including temperature fluctuations, matrix variability, and operator differences, still requires further systematic validation [114,115].

## 8. Conclusions and Perspectives

### 8.1. Detection Characteristics of CSA Technology

Compared with conventional instrumental analysis methods, CSA technology offers several distinct advantages, including ease of operation, rapid detection, low cost, minimal sample pretreatment and suitability for on-site analysis. These attributes make it particularly well-suited for applications such as food spoilage monitoring, adulteration identification and storage quality evaluation. The most distinctive feature of CSA technology lies in its “holistic response” detection mode. Unlike single sensors that target only one specific analyte, CSAs generate characteristic fingerprint patterns through the collective responses of multiple sensing elements, thereby capturing multidimensional chemical information associated with both volatile and non-volatile components in complex food matrices. This cross-responsive mechanism enhances the discrimination of samples with similar compositions or different quality states and reduces the dependence on one-to-one target recognition. Moreover, the inherently visual nature of CSA technology enables detection results to be interpreted by the naked eye or rapidly read via smart terminals, holding significant value for rapid food screening and intelligent packaging applications.

Across the applications reviewed here, CSA systems have progressed from qualitative sample discrimination toward quantitative prediction, freshness grading and time-dependent quality monitoring. The integration of responsive materials, digital imaging and data-driven analysis has further transformed CSA from an isolated colorimetric array into a complete analytical workflow.

### 8.2. Existing Limitations and Challenges of CSA Technology

Despite the considerable progress achieved by CSA technology in food quality detection, numerous challenges remain in its practical implementation. The key challenge, therefore, extends beyond sensitivity alone to the reliability of the complete sensing and analytical workflow.

First, the stability and reproducibility of sensing materials continue to be critical factors affecting CSA performance. Temperature, humidity, illumination, complex food matrices and batch-to-batch variation can cause signal drift, response attenuation and inconsistent array outputs, thereby limiting long-term and continuous monitoring.

Second, the selectivity and anti-interference capability of CSA systems require further improvement. Because CSA relies on holistic fingerprint patterns rather than one-to-one recognition, overlapping responses and background interference from complex food matrices can reduce detection accuracy.

Third, CSA detection outcomes are highly dependent on data analysis algorithms. The detection performance of CSA is substantially influenced by sample size, data quality and model construction methodologies. Although principal component analysis, support vector machines and convolutional neural networks can improve pattern recognition, limited model generalization, difficulties in small-sample learning, poor interpretability and the absence of unified evaluation standards continue to constrain cross-study comparability and model transferability.

In addition, many existing CSA studies remain at the laboratory stage and rely on simulated food systems, limited sample sets or short-term storage experiments. Long-term field performance, interlaboratory reproducibility, scalable manufacturing, economic feasibility and validation under authentic food-supply-chain conditions remain insufficiently investigated. Accordingly, bridging the gap between laboratory research and practical food industry deployment remains a key challenge.

### 8.3. Development Trends and Application Prospects of CSA Technology

In the future, CSA technology will continue to evolve toward high sensitivity, intelligence, miniaturization and integration, playing an increasingly pivotal role in food quality and safety monitoring. However, future progress should be evaluated not only by isolated improvements in sensitivity or classification accuracy, but also by coordinated advances in reliability, comparability, transferability and field performance. Material development should therefore prioritize stable, food-compatible and reproducibly manufactured sensing interfaces that can reduce dye leaching, environmental drift, batch variation and degradation during prolonged use. Meanwhile, standardized procedures for array fabrication, sample handling, headspace exposure, image acquisition, color calibration and performance evaluation are needed to improve measurement comparability across devices, laboratories and food matrices.

At the data and model level, future CSA systems should be developed using more diverse datasets covering different food matrices, sensor batches, imaging devices, environmental conditions and operators. External validation, transfer learning, uncertainty estimation and interpretable analysis will be important for reducing model dependence on specific experimental conditions and improving robustness in unfamiliar scenarios. Multimodal sensing, smart packaging, mobile computing and IoT platforms can further expand the information dimensions and monitoring capabilities of CSA systems; however, their integration should be guided by clearly defined analytical requirements and practical application needs rather than technological complexity alone.

Ultimately, the practical value of CSA technology will depend on systematic validation under authentic food-supply-chain conditions. Prospective studies should evaluate sensor performance throughout food processing, storage, transportation and retail, while also considering manufacturing cost, operational simplicity, regulatory compatibility, maintenance requirements and user acceptance. Progress in these directions would facilitate the transition of CSA technology from laboratory proof-of-concept systems to validated and scalable tools for food-quality screening, process control and supply-chain decision support.

## Figures and Tables

**Table 1:** Comparison of representative sensing materials used in CSA systems for food analysis.

Material Class	Response Mechanism	Advantages	Limitations	Ref.
Chemoresponsive dyes	Molecular structural variation, acid–base reaction, metal–ligand coordination, hydrogen bonding, polarity-dependent response and pH-sensitive structural transformation	Low cost, easy immobilization, intuitive color changes, and suitable for constructing cross-responsive arrays; natural pigments also show better biocompatibility and lower toxicity	Limited selectivity, possible dye leaching, batch variation, and sensitivity to humidity, illumination, pH and storage conditions	[51,52]
Organic nanoparticles	Nanostructure-enhanced optical response, improved dispersion and dual-mode colorimetric/fluorescent readout	Natural-derived, multifunctional, and capable of combining active packaging with freshness indication	Preparation reproducibility, migration safety and long-term stability require further evaluation	[53]
Functional polymers and hydrogels	Polymer conformation change, matrix-assisted dye immobilization, gas adsorption and diffusion-controlled response	Good processability, flexible structure, improved dye retention and gas accessibility	Response rate and sensitivity may depend on matrix swelling, diffusion pathways and environmental humidity	[54]
Immunoassay-related platforms	Antibody/antigen recognition, magnetic preconcentration and nanoparticle-assisted signal amplification	High specificity, low detection limits and suitable for target-specific analysis	Usually require antibodies, multiple assay steps and controlled reaction conditions	[55]
AuNPs	Localized surface plasmon resonance change caused by aggregation, dispersion or target-regulated interactions	Strong visual color change, easy smartphone readout and suitable for rapid screening	Colloidal stability, surface modification, pH, ionic strength and complex matrices may affect reliability	[56]
AgNPs/Ag nanoprisms	Plasmonic color change induced by etching, morphology transformation or aggregation	Rich multicolor response, high visual sensitivity and useful for pattern-based identification	Susceptible to halide concentration, oxidizing/reducing substances and matrix composition	[57]
Nanozymes	Enzyme-like catalytic reaction, such as TMB oxidation, producing amplified colorimetric signals	High stability, low cost, catalytic amplification and better robustness than natural enzymes	Catalytic selectivity, reaction conditions and matrix tolerance still need optimization	[58]
MNPs	Magnetic enrichment, separation and matrix purification before colorimetric readout	Efficient target capture, improved sensitivity and reduced matrix interference	Usually not directly color-generating and often require recognition elements or additional colorimetric steps	[59]
MOFs	Porous adsorption, dye stabilization, VOC enrichment and interface regulation	High surface area, tunable pores, improved dye stability and enhanced VOC response	Synthesis complexity, humidity tolerance and long-term stability need further consideration	[60]

## Data Availability

No new data were created or analyzed in this study.
